# Evaluation the loosening of abutment screws in fluid contamination: an in vitro study

**DOI:** 10.1038/s41598-022-14791-w

**Published:** 2022-06-24

**Authors:** Fei Sun, Wei Cheng, Bao-hong Zhao, Gui-Qiu Song, Zeng Lin

**Affiliations:** 1 Key Laboratory of Implant Device and Interface Science of Liaoning Province, School of Mechanical Engineering and Automation, Northeastern University, Shenyang, 110819 China; 2 Key Laboratory of Oral Diseases of Liaoning Province, School and Hospital of Stomatology, China Medical University, Shenyang, 110002 China

**Keywords:** Biomedical engineering, Mechanical engineering

## Abstract

Screw loosening is one of the most common clinical problems of dental implants. Research on the influencing factors of screw loosening is very important to prevent screw loosening. The purpose of this in vitro study was to evaluate the influence of liquid contamination on the screw loosening. According to the contamination condition, forty-five abutment screws were divided into three groups (n = 15): no contamination, artificial saliva contamination, and mouthwash contamination. The preload and friction coefficient of the abutment screws were recorded. Then, the reverse torque values (RTVs) and settlement were measured after 3.0 × 10^5^ and 6.0 × 10^5^ cycles. The surface wear of the screws was analyzed. Finally, the stress distribution of the abutment screws was calculated by finite element analysis (FEA). The results showed that fluid contamination reduced the friction coefficient, increased the preload, decrease the settlement, improved resistance to screw loosening, and reduced wear on the thread surface. Appropriate antimicrobial lubrication may improve the anti-loosening performance of abutment screws and prevent excessive wear on the threaded surface.

## Introduction

Biomechanical properties are a key factor in the success of dental implants. Despite the high success rate of implantation, some mechanical complications still exist. Abutment screws play a vital role in the connection between the implant and the abutment. Under the long-term influence of the mastication force, abutment screws will experience complications such as loosening or fatigue fracture^1^. One study showed that 26% of abutment screws need to be tightened again after the first year^2^. In another study, the loosening rate of abutment screws was 3.1–10.8% after 5 years^3^. In a clinical study of more than 15 years, the rate of abutment screw fracture was 3.5%^4^. Loose screws will not only cause implant failure but may also cause complications such as gum sensitivity, hyperplasia, and inflammation, which will seriously affect the patient’s daily life^5–7^.

The abutment screw is elastically deformed and elongated due to the torque during the tightening process, and a compression force is formed between the implant and the abutment, which is called the preload^8^. It is generally believed that an increase in the preload helps to improve the stability of the implant-abutment interface^9–11^. When tightening the screw, 90% tightening torque is used to overcome friction, and the remaining 10% is converted into the preload^12^. Friction mainly occurs at the screw-abutment interface and screw-implant interface, and reducing the friction loss between these interfaces enables more torques to be converted into the preload^13^. After the screw is tightened, the preload will be reduced by 2 to 15% due to the settlement effect of the connection interface^14^. Although many methods of increasing the preload are used by reducing the surface friction coefficient, the results of screw loosening are different^15–17^. Therefore, high preload does not mean a good resistance to loosening. The reverse torque values (RTVs) reflects the maintenance of the preload under functional load, which shows the resistance to screw loosening^18^.

In clinical practice, abutment screws will be contaminated by different fluids (saliva, fluorinated artificial saliva, chlorhexidine or blood) due to surgical operations^19,20^. The friction coefficient between the interfaces changes after liquid contamination, which may affect the preload, thereby increasing the risk of screw loosening^21^. Nigro et al. found that a higher preload was produced under wet conditions (inside an implant filled with artificial saliva) than under dry conditions^22^. However, according to Rathe et al., fluid contamination (saliva, blood, or chlorhexidine) did not produce higher preload values^23^. For the research of RTVs, Duarte et al. found that fluoridated artificial saliva can increase the RTVs^24^. Koosha et al. found that only chlorhexidine can increase RTVs, and saliva can decrease RTVs among different liquid contaminations^25^. However, according to Gumus et al., the RTVs of abutment screws decrease after chlorhexidine and saliva contamination^26^. Although some studies have been carried out on the influence of liquid contamination on the abutment screw loosening, there have been various results and no in-depth understanding of the mechanism of screw loosening. Therefore, a study of the screw loosening mechanism under liquid contamination is necessary to improve resistance to the abutment screw loosening. In addition, the research on the influence of fluid contamination on the abutment screw loosening under dynamic load conditions is very limited.

This in vitro study aimed to evaluate the influence of liquid contamination on the screw loosening under static and dynamic conditions. The relationship of preload, settlement, and screw loosening was obtained. The wear of the screw surface was analyzed. A finite element model was also established to obtain the equivalent stress and surface friction stress changes after screw liquid contamination.

## Materials and methods

### Sample preparation

In this study, Morse taper connection dental implants (Ø4.3 × L11 mm), abutments (Ø4.5 × H6.0 mm), and abutment screws (Ø1.5 × L13 mm) were manufactured (WEGO Jericom Biomaterials Co., Ltd., Weihai, China). Forty-five samples were divided into three groups according to the liquid contamination state of the screw (n = 15): control group (NC: no contamination), artificial saliva contamination (SC) group, and mouthwash contamination (MC) group. Artificial saliva was provided by Leagene Biotech. Co., Ltd. (Beijing, China); Mouthwash was obtained by Listerine (Johnson & Johnson Co., Ltd., Shanghai, China). Before the test, the interior of the implant is filled with contaminated liquid through a pipette, and then the abutment and abutment screws were inserted.

### Preload and friction coefficient test

**Figure 1:**
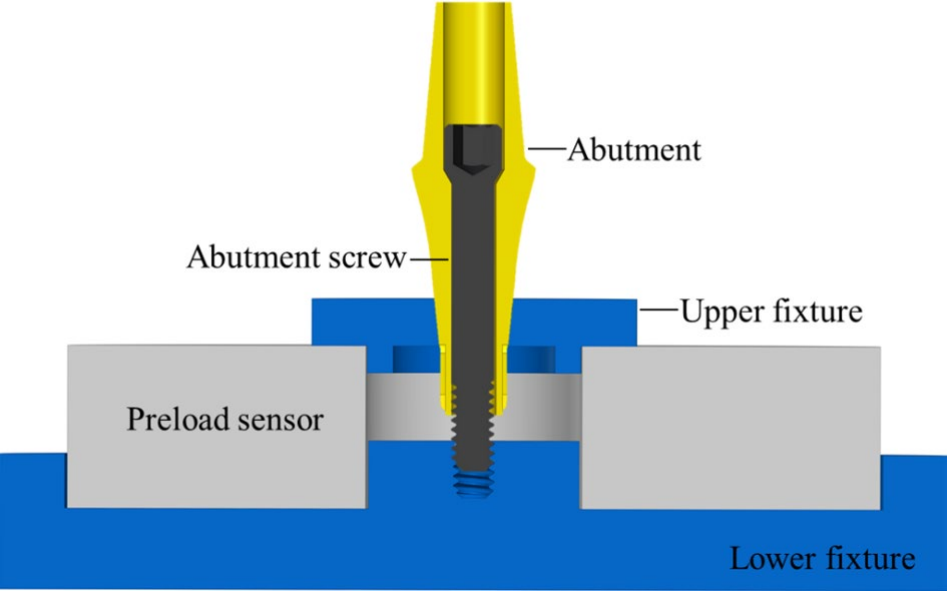
Schematic of the preload test apparatus.

According to ISO16042:2005 "Fastener Torque/Clamping Force Test", the preload (F, N) and friction coefficient (μ) of screws under three surface conditions were measured. A schematic of the preload test apparatus is shown in Fig. 1 (SolidWorks2018, Dassault Système SolidWorks Corp., Concord, MA, USA). The lower clamp was fixed on the workbench, and the preload sensor and the upper clamp were placed. Then, a torque wrench was used to apply the 32 Ncm torque. The preload sensor and torque wrench recorded the values of the preload (F) and tightening torque (T). Five samples were measured in each group. Nisbett^27^ provided the formula for calculating the friction coefficient: (1) T_th_ is the thread torque; (2) T_c_ is the conical torque; and (3) T is the torques sum (T_th_ and T_c_).

1 $$ T_{th} = \frac{{d_{m} }}{2} \times \frac{{L + \left( {\mu \times \pi \times d_{m} \times \sec \alpha } \right)}}{{\left( {\pi \times d_{m} } \right) - \left( {\mu \times L \times \sec \alpha } \right)}} \times F $$

2 $$ T_{c} = \frac{\mu }{3\sin \beta } \times \frac{{D^{3} - d^{3} }}{{D^{2} - d^{2} }} \times F $$

3 $$ T_{{}} = T_{c} + T_{th} $$

where d_m_ represents the pitch diameter (1.37 mm), L represents the pitch (0.35 mm), α represents the half angle of the thread (30°), F represents the preload measured by a sensor (N), μ represents the friction coefficient of the screw, D represents the outer head diameter (2.17 mm), d represents the inner head diameter (1.6 mm), β represents the cone angle (30°), and T represents the tightening torque (Nmm).

### Abutment screw loosening and settlement test

After tightening the three groups of abutment screws to 32 Ncm, the screws were allowed to stand for 10 min, and the RTVs were measured. The average RTVs (T_i_) were recorded. According to Formula (4), torque loss rates (θ) were calculated. Five samples were measured in each group.

4 $$ \theta = \frac{{32 - T_{i} }}{32} \times 100{\text{\% }} $$

where T_i_ represents the RTVs (Ncm).

**Figure 2:**
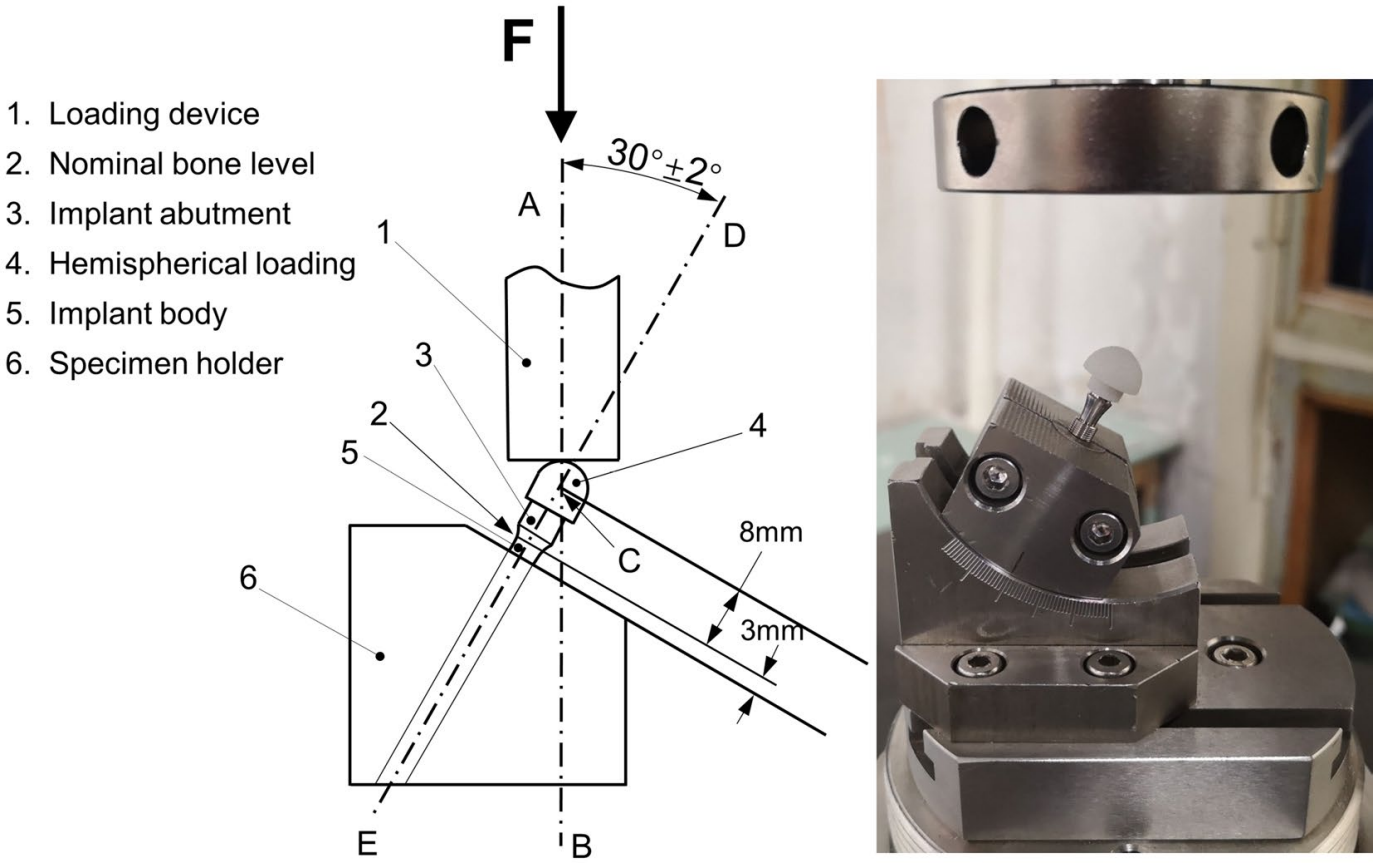
Schematic of the test setup and loading apparatus.

The screw loosening dynamic test was performed according to standard ISO14801:2016^28^, as shown in Fig. 2. The test equipment was a dynamic fatigue testing machine (Care M-3000, Tianjin, China). NC, SC, and MC groups were applied with a dynamic load of 15 Hz (20–200 N), and functional mastication was simulated for 3 months and 6 months with different loading cycles (3.0 × 10^5^ and 6.0 × 10^5^)^16^. After the 3.0 × 10^5^ cycles test was completed, the RTVs (T_3_) were recorded. The liquid was added again after the T_3_ was tested. Then, the screw was retightened for the 6.0 × 10^5^ cycles test, and the RTVs (T_6_) were recorded. In addition, the length of the implant system before and after loading was measured by a spiral micrometer, and the settlement was calculated by the length change. Five samples were measured in each group.

### Morphologic observation

After the dynamic loosening test was completed, the abutment screws were removed and ultrasonically cleaned to remove surface impurities. Scanning electron microscopy with energy dispersive spectroscopy (SEM–EDS) (Ultra Plus, Carl Zeiss AG, Germany) was used to analyze the surface wear of the screws.

### Data analysis

The sample size was calculated using the pre-experimental method (GPower 3.1, A-priori analysis, Germany). Assuming a significance level of 0.05, an effect size of 0.5, and a statistical power of 80%, it is calculated that the required sample size is at least 42 (14 per group). Statistical analyses were performed with SPSS (v20, IBM Corp., USA). Data were analyzed by ANOVA with Fisher LSD test, with P < 0.05 indicating significance.

### 3D-FEA

**Figure 3:**
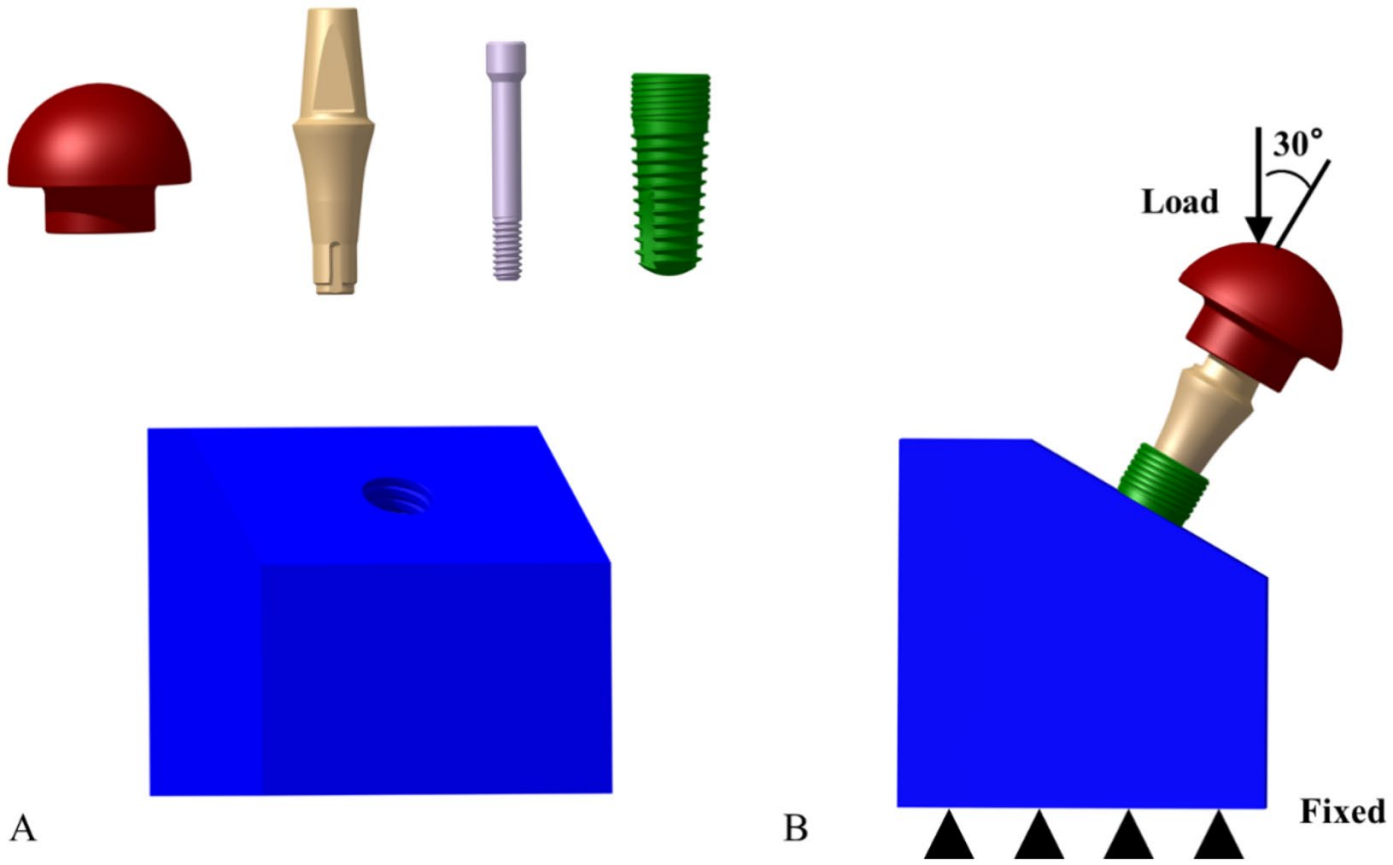
Crown, abutment, abutment screw, implant, and fixture: (**A**) 3D models; (**B**) structure of the finite element model.

To reduce the calculation error, a 3D model of the same size as the implant component was built by the computer-aided design software (SolidWorks2018, Dassault Système SolidWorks Corp., Concord, MA, USA), as shown in Fig. 3. The implant was inserted into a fixture at a 30 degree angle, and the load direction was vertical.

Each part of the model and its material properties were imported into Ansys Workbench software (Ansys Workbench18.0, Swanson Analysis Inc., Houston, PA, USA). Ti6Al4V was used for the abutment and abutment screws, Ti was used for the implants, and stainless steel was used for the crowns and fixtures. The characteristics of the materials are shown in Table 1^29^. The equivalent stress value and frictional stress value were used to assess the distribution of stress among the abutment screws^13,16^. The "frictional contact" was set for screw-implant and abutment-screw, and the "perfect bonding" was set for implant-abutment, implant-fixture, and abutment-crown, and "fixed" was defined for the fixture. According to the measurements performed as described in “Preload and friction coefficient test” section, the three test groups, NC, SC, and MC, had different configurations of the preload and friction coefficient. In addition, a vertical load of 200 N was applied to the implant system (Fig. 3B). The calculated friction stress of the contact part with the screw was used to assess the anti-loosening performance.

**Table 1:** Materials characteristics.

	Elastic modulus (GPa)	Poisson's ratio
Ti	102	0.3
Ti6Al4V	110	0.3
Structural steel	200	0.3

## Results

### Preload and friction coefficient

The preload and friction coefficient of the NC, SC, and MC groups under a torque of 32 Ncm are shown in Table 2. Data analysis revealed significant differences of preload between the SC and NC groups (P = 0.002) and the MC and NC groups (P = 0.001), and no significant difference between SC and MC groups (P = 0.45). The preload of the NC group (325.06 ± 7.71 N) is lower than that of the SC group (367.70 ± 9.83 N) and the MC group (374.07 ± 11.09 N). In contrast, the friction coefficient of the NC group (0.35) is greater than that of the SC (0.3) and MC (0.29) groups.

**Table 2:** Preload and friction coefficient.

Group	F (N)	μ
NC	325.06 ± 7.71	0.35
SC	367.70 ± 9.83	0.30
MC	374.07 ± 11.09	0.29

### RTVs and settlement

Table 3 shows the initial RTVs (T_i_) and the torque loss rate (θ) in the three groups. There were significant differences between the MC and NC groups (P = 0.021), and no significant difference between SC and NC groups (P = 0.214) and the SC and MC groups (P = 0.207). Due to the torque loss during the tightening process, T_i_ was lower than the tightening torque. T_i_ in NC group was 22.82 ± 2.02 Ncm, indicating a 28.69% torque loss. Compared with the NC group, the SC and MC groups had a higher T_i_ and less torque loss, which were SC (T_i_ = 24.16 ± 1.77 Ncm, θ = 24.50%) and MC (T_i_ = 25.52 ± 0.76 Ncm, θ = 20.25%), respectively.

**Table 3:** RTVs (T_i_) and torque loss rates (θ).

Group	T_i_ (Ncm)	θ (%)
NC	22.82 ± 2.02	28.69
SC	24.16 ± 1.77	24.50
MC	25.52 ± 0.76	20.25

**Figure 4:**
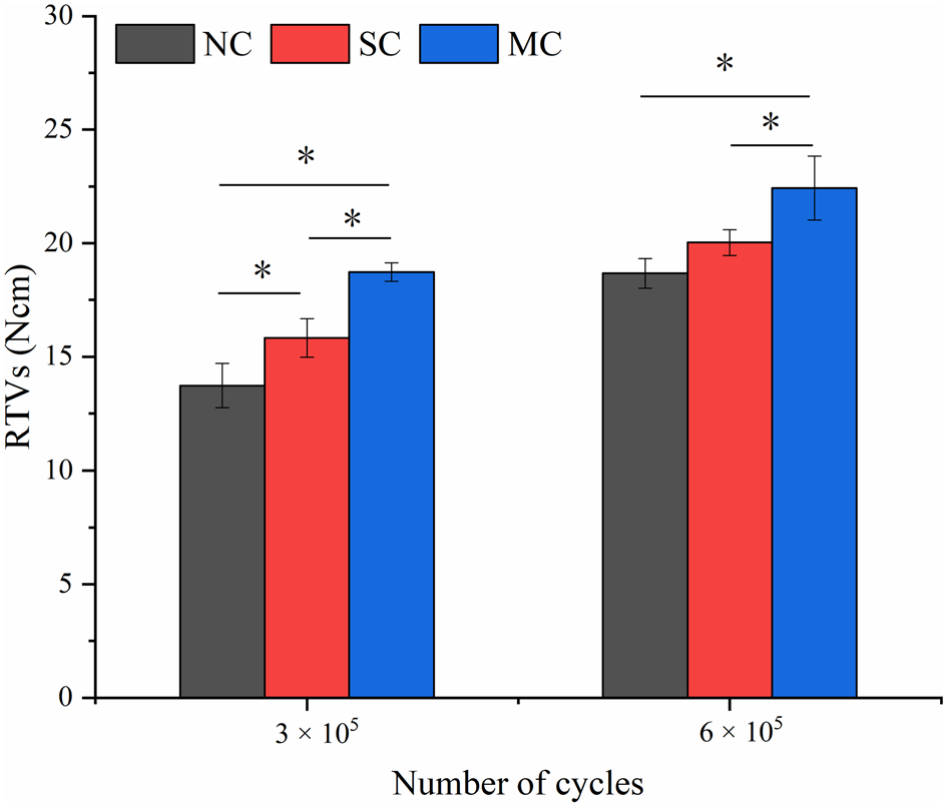
RTVs (T_3_ and T_6_) after cyclic loading in the three groups. *Represents P < 0.05 between groups.

The RTVs (T_3_ and T_6_) after cyclic loading are shown in Fig. 4. T_3_ showed significant differences between the SC and NC groups (P = 0.036), the MC and NC groups (P = 0.001), and the SC and MC groups (P = 0.010). T_6_ showed significant differences between the MC and NC groups (P = 0.008) and the SC and MC groups (P = 0.046), and no significant difference between SC and NC groups (P = 0.202). The RTVs (T_3_ and T_6_) were lower than T_i_ in all groups after cyclic loading. The RTVs in the NC group were less than the SC and MC groups, and the MC group had the highest RTVs. Among the three groups, the T_6_ was greater than the T_3_.

**Figure 5:**
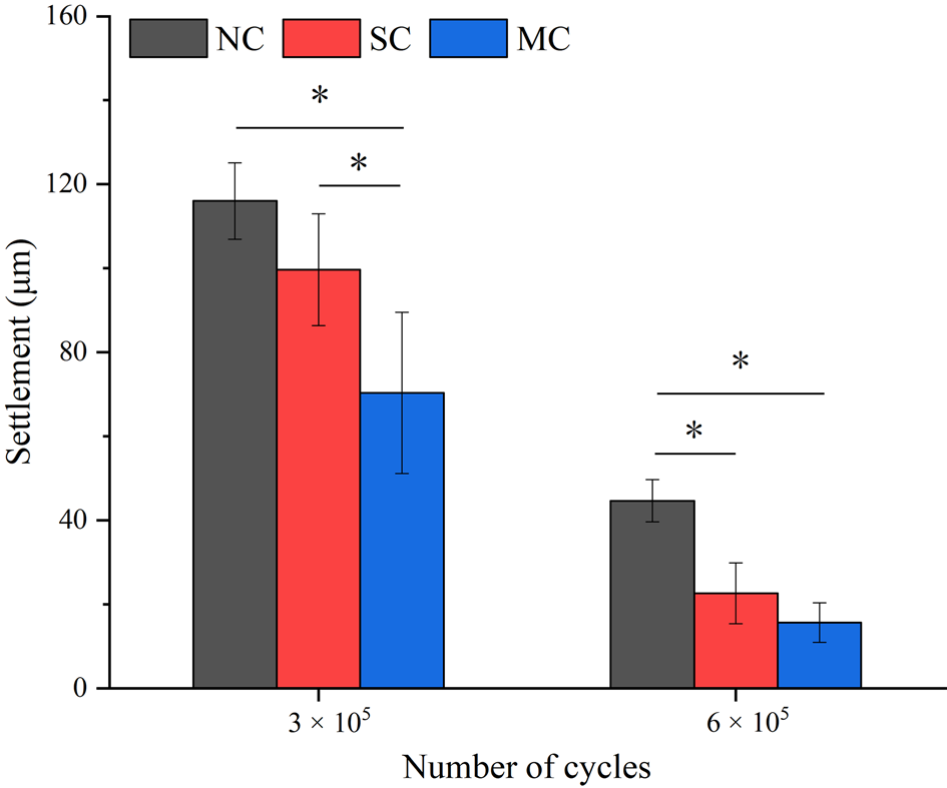
Settlement after cyclic loading in the three groups. *Represents P < 0.05 between groups.

The settlement after cyclic loading are shown in Fig. 5. After the 3.0 × 10^5^ cycles test, settlement showed significant differences between the NC and MC groups (P = 0.008) and the SC and MC groups (P = 0.048), and no significant difference between NC and SC groups (P = 0.217). After the 6.0 × 10^5^ cycles test, settlement showed significant differences between the NC and SC groups (P = 0.003) and the NC and MC groups (P = 0.001), and no significant difference between SC and MC groups (P = 0.188). Among all groups, the NC group had the largest settlement, followed by SC group, and MC group had the smallest settlement.

### SEM investigation

**Figure 6:**
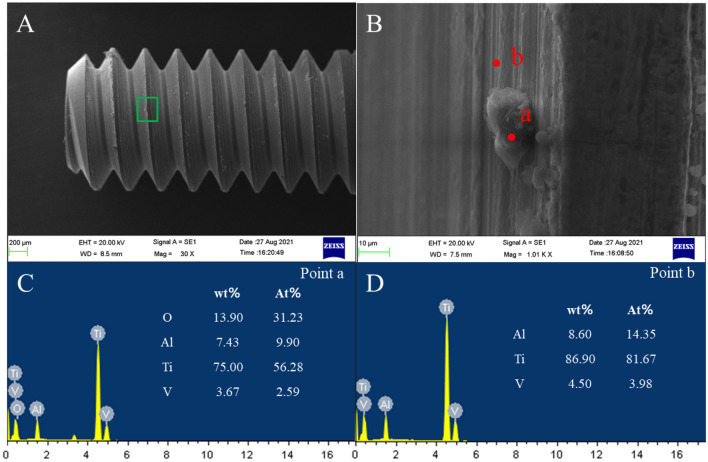
Screw surface wear in the NC group. (**A**) Screw morphology, (**B**) area shown in (**A**), (**C**) EDS at point (a), and (**D**) EDS at point (b).

**Figure 7:**
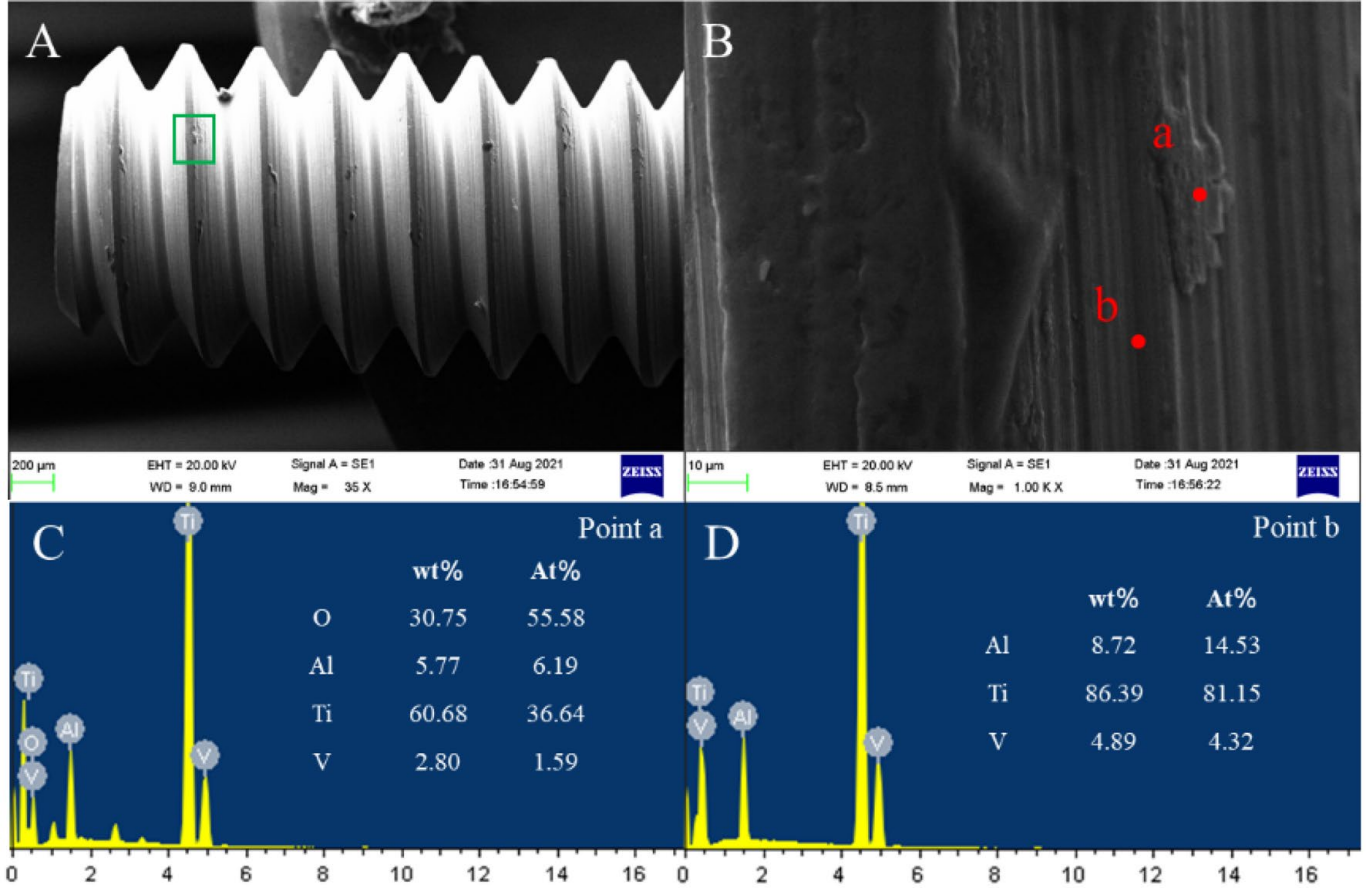
Screw surface wear in the SC group. (**A**) Screw morphology, (**B**) area shown in (**A**), (**C**) EDS at point (a), and (**D**) EDS at point (b).

**Figure 8:**
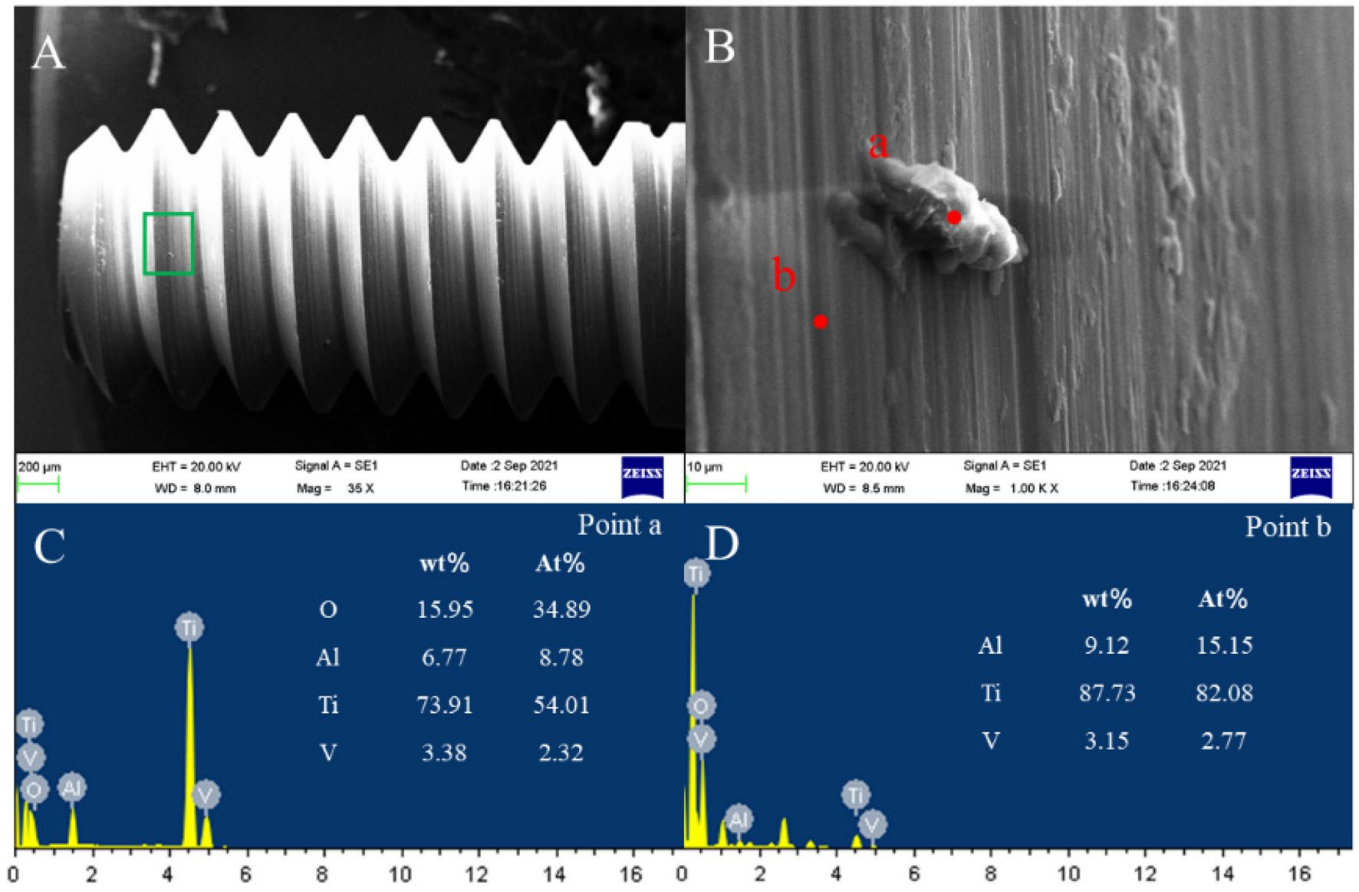
Screw surface wear in the MC group. (**A**) Screw morphology, (**B**) area shown in (**A**), (**C**) EDS at point (a), and (**D**) EDS at point (b).

Figure 6 shows the SEM and EDS results of the abutment screw surface in the NC group. As shown in Fig. 6A, wear can be clearly observed on the thread surface of the NC group, and the wear is uneven. Many small wear debris and local accumulation of wear debris were found in Fig. 6B. Figure 6C,D are the EDS analysis results of point (a) and point (b) in Fig. 6B, respectively. Compared to point (b), point (a) shows an O-element peak. Figure 7 shows the SEM and EDS results in the SC group. As shown in Fig. 7A, there is only a small amount of wear debris on the thread surface. In addition, the plastic flow phenomenon is observed in Fig. 7B. Figure 7C,D are the EDS analysis results of point (a) and point (b) in Fig. 7B, respectively. Compared to point (b), point (a) also shows an O-element peak. Figure 8 shows the SEM and EDS results in the MC group. As shown in Fig. 8A, the wear on the screw surface of the MC group was similar to that of the SC group, with only slight wear on the surface. In Fig. 8B, in addition to the observed debris, a slight plastic flow is also observed. Figure 8C,D are the EDS analysis results of point (a) and point (b) in Fig. 8B, respectively. EDS analysis found that the point (a) contains an O-element peak.

### 3D-FEA

**Figure 9:**
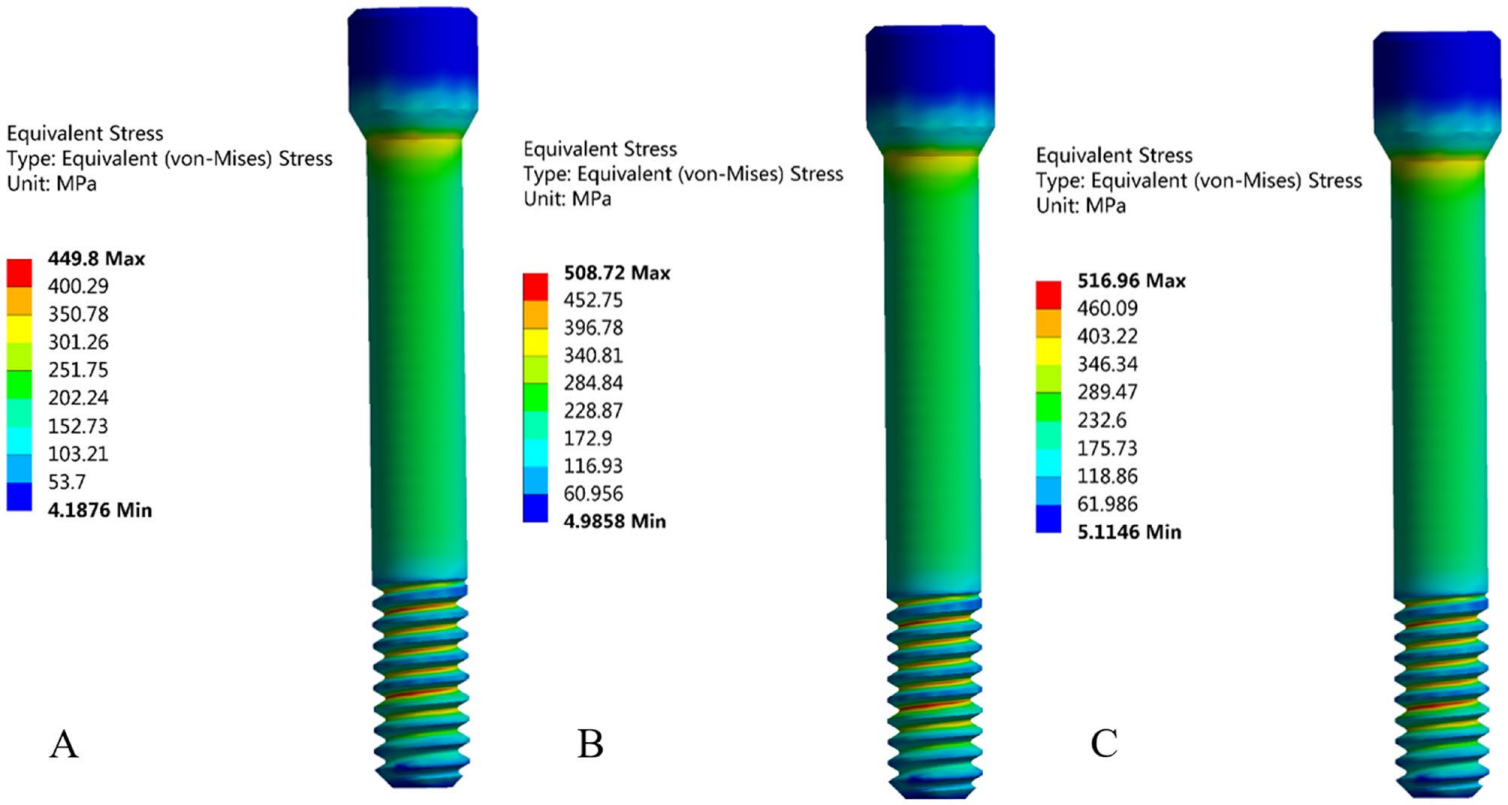
Equivalent stress distribution of the abutment screws. (**A**) NC group, (**B**) SC group, and (**C**) MC group.

**Figure 10:**
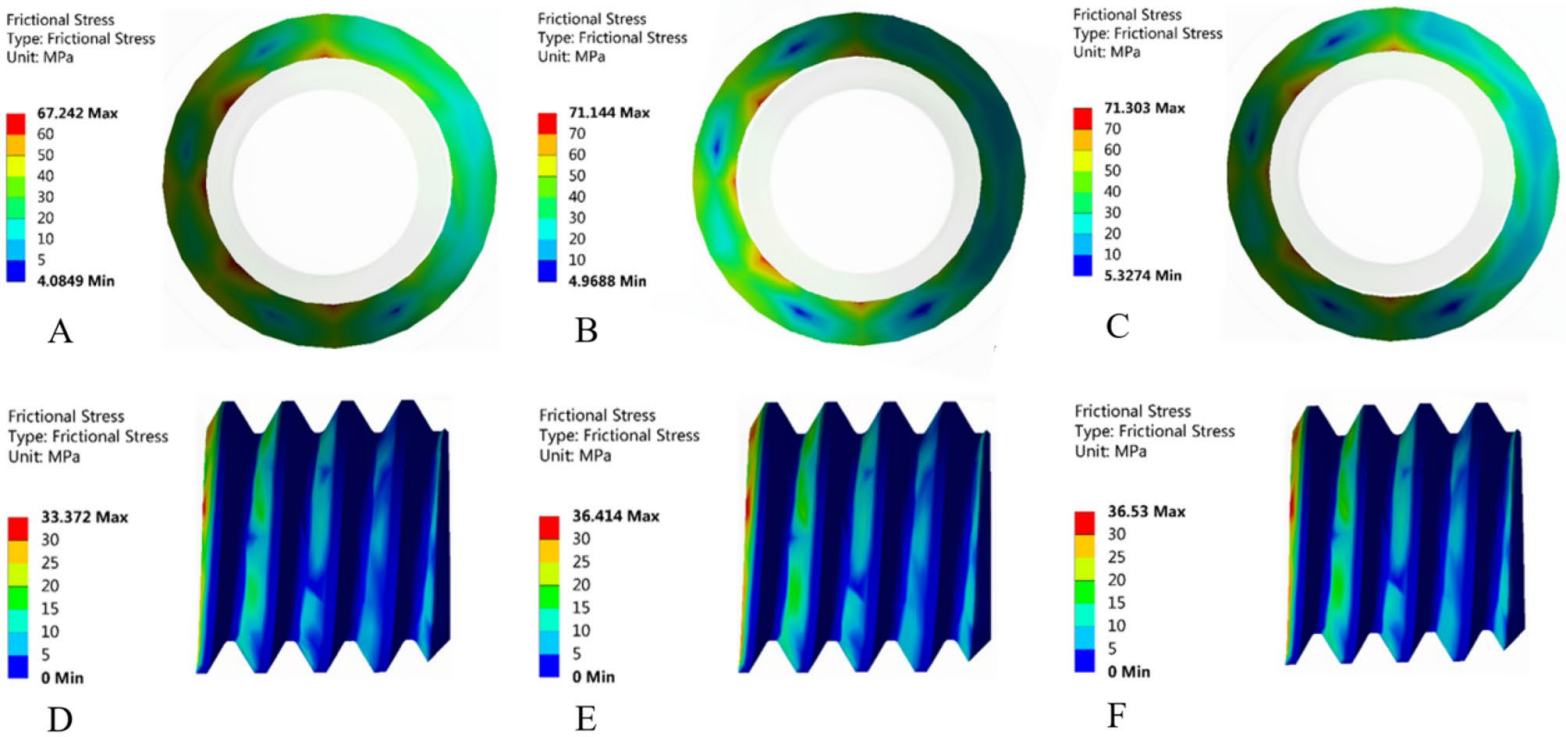
Frictional stress distribution of the screw taper surface and thread surface. (**A**,**D**) NC group, (**B**,**E**) SC group, and (**C**,**F**) MC group.

When the external load was 200 N, Fig. 9 shows the equivalent stress distribution results of the three groups of abutment screws. The equivalent stress of the screw was closely related to the surface condition, and the stress concentration mainly occurred at the thread position and the taper connection position of the screw head. The abutment screws of the NC group showed the smallest equivalent stress (Fig. 9A, 449.8 MPa), followed by the screws of the SC group (Fig. 9B, 508.72 MPa), and the abutment screws of the MC group showed the largest equivalent stress (Fig. 9C, 516.96 MPa). The frictional stress of the contact area with the abutment screw is shown in Fig. 10. Frictional stress occurred on the taper surface between the screw and the abutment and the threaded surface between the screw and the implant. The friction stress distribution of the screw taper surface and thread surface of the NC group is shown in Fig. 10A,D. Compared with the NC group, the friction stress of the SC and MC groups in the contact area with the abutment increased by 5.8% (Fig. 10B) and 6% (Fig. 10C), respectively, and the friction stress in the contact area with the implant increased by 9.1% (Fig. 10E) and 9.5% (Fig. 10F), respectively.

## Discussion

The present experiment investigated the influence of liquid contamination on the connection stability of the implant system. The results showed that the implant system after liquid contamination showed significant differences in the screw loosening and surface wear.

The preload is generated by the tensile deformation during screw tightening, and its optimal range is 60% ~ 70% of the yield strength of the screw^11^. The liquid-contaminated abutment screw produced a higher preload and a lower friction coefficient. Studies have shown that the probability of reaching the best range in a well-lubricated environment is 54%, while in a dry environment, it is only 0.02%^30^. During the tightening of the screw, sliding friction occurred between the screw thread and the internal thread of the implant, but this friction was not evenly distributed. The wet medium can eliminate most of the shear forces generated inside the thread^31^ and reduced the friction coefficient of the thread surface, which reduced the energy loss due to friction so that more tightening torque was converted into preload. Previous studies have also confirmed that a lower friction coefficient will lead to a higher preload^13,32,33^.

Although liquid contamination increases the screw preload, it is more important that the preload remains stable rather than dropping rapidly^29^. Therefore, the RTVs of liquid-contaminated screws before and after loading was evaluated. The T_i_ of the SC and MC groups that have been liquid-contaminated are larger than those of the NC group, consistent with previous studies^22,25^. This is because the high preload made the threaded connection tighter. In addition, microscopically, the thread surface was rough, and the embedment relaxation occurred under the state of force^34^. The liquid reduced the embedment relaxation of the thread surface, thereby reducing preload dropped^22^.

After a cyclic load of 3 × 10^5^ cycles, T_3_ of the three groups decreased, which was lower than their respective T_i_. The vibration generated by the external load intensified the slippage of the threaded contact surface, and the settlement effect occurred at the implant-abutment interface^35^, resulting in reduced preload and more torque loss. The two groups of liquid contamination showed better anti-loosening performance after dynamic cyclic loading. The larger the preload of the screw is, the smaller the fretting between the parts when subjected to external force, so that the torque is not easy to lose^10^. In addition, the abutment settlement was reduced due to the liquid inside the implant (Fig. 5), thus reducing the risk of insufficient tension of the screw^36^. After a cyclic load of 6 × 10^5^ cycles, T_6_ of the three groups has a certain increase, and the trend is similar to that of 3 × 10^5^ cycles. This is because after 3 × 10^5^ cycles, the originally rough contact surfaces were squeezed and smoothed out by each other, and more abutment settlement had occurred. When a cyclic load of 6 × 10^5^ cycles was performed, the taper surfaces had less settlement, and thread surfaces were more tightly bonded, resulting in an increase in T_6_^37^. Therefore, after a certain number of cycles, loosening and retightening helped prevent the screw from loosening^38^.

In addition, there are differences in the lubricating effects of the two liquids. The RTVs of the MC group were higher than those of the SC group. The main factors affecting the lubrication effect of the two liquids are the viscosity, concentration, and composition of the liquid^26^. The viscosity will affect the flow of liquid on the metal surface and affect preload and settlement^39^. Therefore, the RTVs were improved to varying degrees.

In the process of the dynamic fatigue cycle, due to the existence of fretting, the screw surface will inevitably wear. The mechanism of wear may be abrasive wear, adhesive wear, oxidation wear, fatigue wear, etc.^40^. This can cause surface deformation, preload loss, and even loosening of screws. Effective liquid lubrication can protect the thread surface from excessive wear and prevent the aggravation of fretting damage^26^. The wear mainly occurred in the top area of the thread, mainly abrasive wear and oxidation wear, which was consistent with previous research^29,34,41^. Liquid lubrication increased the preload of the screw and reduced abutment settlement, thereby reducing the relative sliding of the threaded surface and surface wear^42^.

The 3D-FEA results can explain the reason for the increase in the RTVs of the liquid-contaminated group. The equivalent stress and friction stress of the screw increased with increasing preload. This made the bonding between the contact surfaces closer, and the screw reached the appropriate stress value^11^. The increase in friction stress indicated that a greater friction force was generated between the contact surfaces, and relative sliding did not easily occur between the contact surfaces^40^. Therefore, the RTVs are greater, and the system stability will be improved. In addition, comparing the contact stress between screw-implant and screw-abutment, it was found that the friction stress in the contact area with the abutment was greater than the friction stress in the implant. The reason may be that the contact area of the abutment was small and there was a concentration of stress, which was not as good as the implant-screw threaded contact for dispersing the friction stress.

This study still has some limitations. For example, the FEA results are only under static conditions, which cannot reflect the dynamic screw loosening process. The types of contamination assessed are few and single, because screws are contaminated by multiple substances in the oral environment. Only screw loosening behavior was studied, but the effects of liquid contamination on aging and corrosion were not considered. Additionally, loading conditions in vivo are different from in vitro studies, so more complex loading states need to be tested. The Morse taper connection was used in this study, and other connected implant systems need to be studied in the future.

## Conclusion

In summary, liquid contamination on the abutment screw surface can increase the preload, reduce the friction coefficient, and reduce the settlement, thereby improving the resistance to loosening of the screw and reducing the wear of the screw surface. Therefore, antibacterial solutions or gels with lubricating properties may improve the long-term stability of implant systems.

## Data Availability

The datasets generated during and/or analysed during the current study are available from the corresponding author on reasonable request.
